# Multiple Time Series Fusion Based on LSTM: An Application to CAP A Phase Classification Using EEG

**DOI:** 10.3390/ijerph191710892

**Published:** 2022-09-01

**Authors:** Fábio Mendonça, Sheikh Shanawaz Mostafa, Diogo Freitas, Fernando Morgado-Dias, Antonio G. Ravelo-García

**Affiliations:** 1 Interactive Technologies Institute (ITI/LARSyS and ARDITI), 9020-105 Funchal, Portugal; 2 Higher School of Technologies and Management, University of Madeira, 9000-082 Funchal, Portugal; 3 Faculty of Exact Sciences and Engineering, University of Madeira, 9000-082 Funchal, Portugal; 4 NOVA Laboratory for Computer Science and Informatics, 2829-516 Caparica, Portugal; 5 Institute for Technological Development and Innovation in Communications, Universidad de Las Palmas de Gran Canaria, 35001 Las Palmas de Gran Canaria, Spain

**Keywords:** CAP A phase, Genetic algorithm, information fusion, Particle Swarm Optimization, LSTM

## Abstract

The Cyclic Alternating Pattern (CAP) is a periodic activity detected in the electroencephalogram (EEG) signals. This pattern was identified as a marker of unstable sleep with several possible clinical applications; however, there is a need to develop automatic methodologies to facilitate real-world applications based on CAP assessment. Therefore, a deep learning-based EEG channels’ feature level fusion was proposed in this work and employed for the CAP A phase classification. Two optimization algorithms optimized the channel selection, fusion, and classification procedures. The developed methodologies were evaluated by fusing the information from multiple EEG channels for patients with nocturnal frontal lobe epilepsy and patients without neurological disorders. Results showed that both optimization algorithms selected a comparable structure with similar feature level fusion, consisting of three electroencephalogram channels (Fp2–F4, C4–A1, F4–C4), which is in line with the CAP protocol to ensure multiple channels’ arousals for CAP detection. Moreover, the two optimized models reached an area under the receiver operating characteristic curve of 0.82, with average accuracy ranging from 77% to 79%, a result in the upper range of the specialist agreement and best state-of-the-art works, despite a challenging dataset. The proposed methodology also has the advantage of providing a fully automatic analysis without requiring any manual procedure. Ultimately, the models were revealed to be noise-resistant and resilient to multiple channel loss, being thus suitable for real-world application.

## 1. Introduction

The sleep macrostructure can be divided into Rapid Eye Movement (REM) and Non-REM (NREM) periods. Moreover, in order to examine the sleep microstructure during NREM, the electroencephalogram (EEG) Cyclic Alternating Pattern (CAP) concept can be used. This pattern is composed of an initial phase of brain activation, named the A phase, followed by a period of return to the background activity, denoted the B phase. Both phases must have a duration between two and 60 s to be considered valid, and a B phase must be bounded by two A phases. Two or more successive CAP cycles define a CAP sequence [1,2,3].

CAP was shown to be related to the formation, consolidation, and disruption of the sleep macrostructure, working as a measure of the brain’s effort to maintain sleep [3,4,5]. It was also acknowledged as an EEG marker of sleep instability. In addition, a temporal relationship exists between CAP, behavioral activities, and autonomic functions [5]. As a result, the CAP was found to be linked with the incidence of several sleep disorders, including insomnia [6], Nocturnal Frontal Lobe Epilepsy (NFLE) [7], sleep apnea [8], periodic limb movements [9], and idiopathic generalized epilepsy [10]. 

Therefore, the employment of CAP analysis by the sleep centers can lead to significant advances in the diagnosis and characterization of sleep quality. However, the introduction of CAP analysis as a regular clinical practice faces some obstacles, namely (a) the time required for manually scoring whole night polysomnography (the gold standard for sleep analysis [11]) due to the large amount of information produced during whole night EEG recording, (b) the combination of information from different sensors or channels, (c) the need for qualified personnel to perform the manual scoring, and (d) the fair inter-scorer specialist agreement, that varies from 69% to 78% [12]. Therefore, manual scoring is considerably problematic, as the process is unpractical and prone to misclassifications. In addition, it was also observed that CAP is a global EEG phenomenon comprising extensive cortical areas, suggesting that the A phases could be visible on all EEG channels [1]. However, the state-of-the-art works on proposed methodologies for automatic A phase analysis perform the examination using only one EEG channel (usually with one monopolar derivation). Although this approach can lead to less complex models, it is also reductive and restrictive since a large amount of information coming from the other channels is discarded, disregarding at the same time the fact that the A phase activity can occur over multiple cortical areas. For these reasons, the development of algorithms for automatic CAP analysis with information fusion, besides being desirable, is the focus of this work. 

Information fusion technologies enable the combination of information from multiple sources to unify and process data. These technologies can thus transform the information from different sources into a representation that provides adequate support for automatic analysis [13]. There are two fundamental methods to process data from multiple sources. The first, known as centralized fusion, employs a fusion center to receive and process information from different sources. In the second (known as distributed fusion), differently from the first method, each source provides a local estimation from its measured data to the fusion node, which then performs the fusion. The first method can attain optimal performance. However, the second has higher robustness, a relevant characteristic mainly when biomedical sensors, such as EEG, are used since these can be easily contaminated with noise or lose contact [14]. 

Information fusion was applied successfully in numerous fields [15]; among these, body sensors’ analysis attained significant developments with revolutionary applications in healthcare and fitness examination [16]. The fusion of information from multiple sources reduces noise effects, improves the robustness against interference, and reduces ambiguity and uncertainty, seeing that using an individual source of information is often insufficient to provide a reliable examination. 

The hierarchy of information fusion can be divided into three main levels. First is the data level fusion techniques, such as Kalman filter and averaging methods, operating at the lowest level of abstraction to combine raw data from multiple sources [17]. The second performs the fusion at the feature level, where feature sets extracted from different data sources are combined to create a new feature vector. The last one is carried out at the decision level and deals with the selection (or creation) of a hypothesis from the set of hypotheses and is usually performed by fuzzy logic, Bayesian inference, classical inference, or heuristic-based schemes (such as majority voting) [16]. The data level and feature level fusion are generally done before classification or any hypothesis selection or creation of the data. Afterward, the decision level fusion is done.

Fusion-based models are a suitable choice when combining multiple information sources. These models might lead to better performance, particularly when compared to the use of single information source models. In this view, A phase classification is a proper problem for fusion-based approaches. Therefore, it was hypothesized in this work that the fusion of multiple EEG channels could provide more relevant information for the automatic A phase classification when compared to single-channel models. In other words, the main goal of this work is to develop an automatic classifier for the A phase assessment based on the signals from multiple EEG channels.

The key novelties of this work can be summarized as follows:

- Proposal of a novel method for information fusion based on a deep learning model responsible for extracting the features, performing the feature level fusion, and performing the classification. The optimization algorithm tuned the structure of the classifier. Hence, all the fusion and classification procedures were optimized and executed automatically by the deep learning model, which learned the relevant patterns directly from the data.
- Independent evaluation of two optimization algorithms for finding the optimal structure of a deep learning classifier. Optimizing deep learning models is a well-known difficulty in machine learning since the simulations are usually slow. Therefore, there is a need to study suitable algorithms to haste this process.
- Combined examination of subjects free from neurological disorders and subjects with a sleep-related disorder using information (i.e., the signal) from multiple EEG channels to assess the CAP A phases. The state-of-the-art standard is only to examine one channel for the analysis, which is contrary to the specification of the CAP protocol, where the examination should preferably be carried out over multiple channels [1].
- Development of systems tolerant to noise (until a signal-to-noise ratio of 0 dB) and able to handle the loss of 66% of the information, i.e., loss of two channels.

It is essential to highlight here that the CAP A phase assessment was used as an example of applying the proposed fusion of multiple time series. In other words, the suggested approach was developed to be generic and thus could be applied to further research and industry applications.

The article has the following organization: the employed materials and methods are presented in Section 2; the model’s performance is evaluated in Section 3; a discussion of the obtained results is carried out in Section 4; the paper is concluded in Section 5.

## 2. Materials and Methods

The developed model estimates the CAP A phases, in a second-by-second assessment, by examining the preprocessed signals from multiple EEG channels. Those signals were fused by the deep learning classifier that performed the automatic feature extraction and classification. Specifically, distributed fusion was employed in this work since it is suitable when the sources of information come from similar sensors [18]. Each EEG channel was fed to one Long Short-Term Memory (LSTM), which was used to extract features from each signal. Afterward, the fusion node concatenated the extracted features to produce the fused feature vector (feature level fusion [16]) employed to perform the A phase classification. 

The deep learning classifiers’ structure and/or hyperparameters are usually selected through an experimental search (usually a grid search), which performs an exhaustive evaluation of multiple combinations of parameters. However, this approach requires significant time and computational resources, which can be impracticable for deep learning models [19]. Two heuristic-based algorithms, namely, Genetic Algorithms (GA) and Particle Swarm Optimization (PSO), were used in this work, alternatively to the grid search approach, to find the optimal structure, number of channels, and hyperparameters of the models [20]. These types of methods were selected as they have been proven in state-of-the-art to be capable of solving optimization-based problems in different domains [21,22], such as analog filter design [23], task allocation and scheduling [24], route planning [25], image classification [26], and design and planning of production systems [27]. Therefore, two models were developed to perform the channel fusion of EEG channels for the CAP A phase assessment. One was tuned by a GA and the other by the PSO algorithm. It is also intended to study the optimization algorithms’ characteristics to determine what can lead to the best performance. 

The classifier’s output was post-processed to reduce the misclassification, and the model’s performance was assessed. The pseudocode of the developed model is presented in Algorithm 1. The code developed for this work was made open-source, available in a GitHub repository (https://github.com/Dntfreitas/EA_Time_Series_Fusion_Optimizer (accessed on 26 August 2022)).
**Algorithm 1** Pseudocode for the experimental procedure.
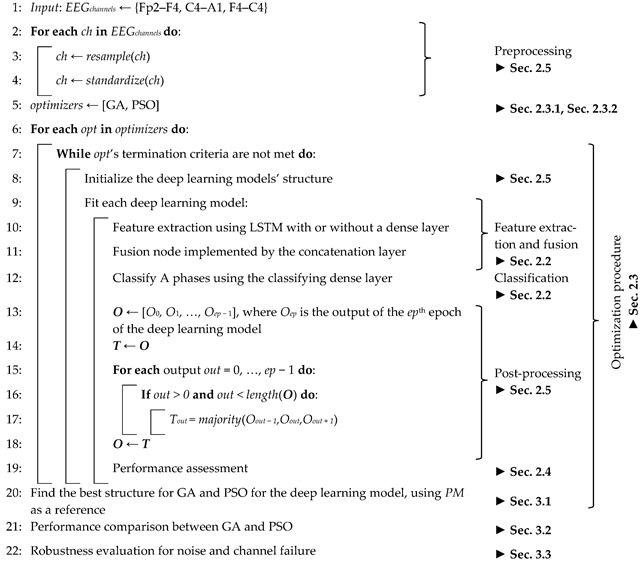



### 2.1. Studied Population

Recordings from the CAP Sleep Database [1,28] were selected to develop the model. This database is publicly available and has annotations provided by sleep experts regarding the A phase occurrence and duration. A total of 16 subjects were examined, using the signals from eight subjects free of Neurological Disorder (FND) and eight subjects with a sleep-related disorder, identified as Sleep Disorder Patients (SDP), to provide a broader representation of the general population. NFLE was chosen to be the studied disorder since the epileptic manifestations are likely to act as a sub-continuous “internal noise” that can induce a substantial growth of all CAP-related parameters, reflecting the degree of sleep instability [7]. According to our best knowledge, no state-of-the-art work examined a combination of normal subjects and subjects with NFLE in the task of automatic classification of CAP A phases.

Therefore, the considered population was composed of eight normal subjects (reference for normal sleep quality) and eight subjects prone to having poor sleep quality. The population was composed of 11 females and five males, which was either equal to or higher than the works available in the state-of-the-art performing the CAP A phase analysis. A summary of the demographic characteristics of the studied population is presented in Table 1. The average total sleep time of the studied population was 27,761.25 s, ranging from 22,230.00 to 33,210.00 s, with a standard deviation of 3197.07 s. The number of one-second epochs related to an A phase was 64,003.

Relevant information for the CAP analysis is present in EEG bipolar and monopolar derivations since the CAP is a global EEG phenomenon comprising broad cortical areas [1]. Mariani et al. [29] reported that CAP analysis usually uses only the signal from one monopolar derivation (either C4–A1 or C3–A2). However, such methodology is prone to have many false positives (identified A phases) as many activations correspond to changes in amplitude and/or frequency on the central lead but are regular EEG rhythms on the others. Therefore, CAP scoring should be performed by scoring multiple channels [29].

In that view, the goal is to use as many derivations as possible while keeping the model’s complexity feasible to be used in the currently available hardware. It was observed that the state-of-the-art works examined either the F4–C4 channel or one monopolar derivation (C4–A1 or C3–A2). Nevertheless, Terzano et al. [1] indicated that all bipolar derivations could adequately detect the A phases; consequently, the Fp2–F4 was also examined in this work. Hence, the three examined deviations are Fp2–F4, F4–C4, and C4–A1.

### 2.2. Classification and Channel Fusion

Most methods proposed in state-of-the-art A phase detection employ classification with features created by the researchers. Nevertheless, significant domain-specific knowledge is required for the feature creation process, and it is becoming increasingly challenging to discern a new set of features that can perform better than the methods already reported in the state-of-the-art. Additionally, there is a need for feature sorting, which does not guarantee a performance improvement [20,30]. These complications can be surpassed by a deep learning model, which can automatically learn the relevant patterns from the input signal. However, a significant gap in the state-of-the-art methods regarding deep learning applications for CAP analysis was identified.

CAP phases have a strong temporal dependency that can be captured by recurrent neural networks, e.g., LSTM [31], and the activity can be measured in different EEG channels. Therefore, a novel approach was followed in this work where the information from multiple EEG channels was fused by a proposed deep learning channel fusion methodology, composed of LSTM, concatenation, and fully connected (dense) layers.

Each LSTM layer comprises memory cells that sequentially process the input and preserve their hidden state through time [32]. Each cell is controlled by three gates. The input gate (*I*) defines the flow of activations into the cell, while the output gate (*O*) controls the flow of activations to the remaining network. The forget gate (*F*) is responsible for adaptively resetting the cell’s state. For the time step *t* and cell *c*, these operations are defined as [33].

(1) $$F_{c}^{\left(t\right)}=\sigma \left(\underset{j}{\sum }U_{c,j}^{F}x_{j}^{\left(t\right)}+\underset{j}{\sum }W_{c,j}^{F}h_{j}^{\left(t-1\right)}+b_{c}^{F}\right)$$

(2) $$I_{c}^{\left(t\right)}=\sigma \left(\underset{j}{\sum }U_{c,j}^{I}x_{j}^{\left(t\right)}+\underset{j}{\sum }W_{c,j}^{I}h_{j}^{\left(t-1\right)}+b_{c}^{I}\right)$$

(3) $$O_{c}^{\left(t\right)}=\sigma \left(\underset{j}{\sum }U_{c,j}^{O}x_{j}^{\left(t\right)}+\underset{j}{\sum }W_{c,j}^{O}h_{j}^{\left(t-1\right)}+b_{c}^{O}\right)$$

where *σ* is the sigmoid function given by *σ* (*α*) = 1/(1 + e^−*α*^), ***x***^(*t*)^ is the input vector, ***U*** are the input weights, ***W*** are the recurrence weights, and ***b*** are the bias. The network’s output, ***h***, is given by [33] $h_{c}^{\left(t\right)}=tanh\left(s_{c}^{\left(t\right)}\right)o_{c}^{\left(t\right)}$, where *tanh* is the hyperbolic tangent function calculated as *tanh*(α) = 2*σ*(2α)–1, and *s*^(*t*)^ is the cell’s internal state, updated by

(4) $$s_{c}^{\left(t\right)}=f_{c}^{\left(t\right)}s_{c}^{\left(t-1\right)}+i_{c}^{\left(t\right)}tanh\left(\underset{j}{\sum }U_{c,j}x_{j}^{\left(t\right)}+\underset{j}{\sum }W_{c,j}h_{j}^{\left(t-1\right)}+b_{c}\right)$$

An LSTM layer can examine the data sequence in only one direction (conventional LSTM model) or two directions, denoted as Bidirectional LSTM (BLSTM). Although the BLSTM models use more parameters when compared to the conventional LSTM models, these models can likely find more relevant patterns in the fed data.

Each LSTM cell receives a time step of data with duration *D*, composed of *I* input points. The optimization algorithm chose the type of LSTM, the number of channels, *n*, number of time steps, *T*, and the number of LSTM layers (stacked if more than one). Each cell has multiple hidden units, and the total number of hidden units, *H*, of the last cell, defines the output of the LSTM layer (the epoch’s data fed to the last cell corresponds to the database label for the current evaluated epoch). When two LSTM layers were stacked, the sequence of vectors of the first layer was returned to the second layer, whose last cells’ outputs defined the output.

The LSTM layers’ output the features ***h***_1_, ***h***_2,_ …, ***h****_n_* that were automatically crafted from each input channel. These features were then transformed to ***f*** = [***f***_1_ [*h*_1_(1), *h*_1_(2), …, *h*_1_(*H*)], ***f***_2_ [*h*_2_(1), *h*_2_(2), …, *h*_2_(*H*)], …, ***f****_n_* [*h_n_*(1), *h_n_*(2), …, *h_n_*(*H*)]] by the concatenation layer, where ***f***_1_, ***f***_2,_ …, ***f****_n_* are either the outputs of the LSTM (***h***_1_, ***h***_2,_ …, ***h****_n_*), or are the dense layers’ transformations of the LSTM outputs, according to the decision of the optimization algorithm. These channels were fused, at the feature level, by the concatenation layer, which merges all the features into a sequence ***f***, i.e., the input of the fusion node is the set of features ***h***, and the output is ***f***. If a dense layer was used to transform the LSTM layer’s outputs; then a second dense layer (with the same configuration as the first dense layer) was used to transform the concatenation layer’s output.

In the end, the softmax function, given by softmax(*α*) = e*^α^*/$\underset{j}{\sum }e^{\alpha_{j}}$, was used by a fully connected layer to normalize the output. Finally, the binary classification output was obtained by applying the max operation.

### 2.3. Optimization Procedure

Two optimization algorithms (i.e., GA and PSO) were studied to find the best classifier structure for the A phase assessment, evaluating an encoding array. The GA was selected since it is one of the most commonly used algorithms for complex design optimization problems, using Darwinian principles of biological evolution [20]. On the other hand, PSO methodology is based on information sharing, such as what occurs in nature in the flocks of birds and schools of fish, and this algorithm was selected since it has considerable flexibility and is capable of finding the globally best solution in complex (possibly multimodal) search spaces [34].

These stochastic algorithms were used in this work as an alternative to the conventional grid search, which is considered unfeasible, especially when many parameters must be tuned [20]. A brief description of applied GA and PSO is presented in Section 2.3.1 and Section 2.3.2, respectively, where the goal is to find the solution that maximizes the Performance Metric (*PM*).

#### 2.3.1. Genetic Algorithm

GA is a metaheuristic algorithm that has previously shown to be capable of finding an improved solution over time by replicating the best solutions from generation to generation and producing offspring from these solutions [35].

For this work, the algorithm was initialized with a random individual generation, using mutation and crossover operators over a defined number of generations to reach a solution, which optimized the model to a given metric. A pseudocode of GA is shown in Appendix A (Algorithm A1).

Coded chromosomes were employed to characterize the population *P* = [*p*_1_, *p*_2_, …, *p_z_*], where *z* is the size of each generation, *g*. Each *p* was decoded using a decoding table (see Table A1 in Appendix B), and the selected *PM* assessed the quality of the solution (fitness assessment). The algorithm stopped if the maximum number of generations, *G*, or if the patience value, *Pa*, (number of consecutive generations that the algorithm did not produce an improved solution) reached the maximum patience, *Pa_max_*. The initial population of *P* was randomly generated and then sorted according to the performance of each chromosome. Afterward, a new cycle started to create the offspring population, *Q*, with size *z*. According to the crossover probability, each new member of the offspring population, *q*, was created either by a two-point crossover operation between two different elements randomly chosen from *P* or by cloning the most fitted element selected from a tournament of two. In the two-point crossover operation, each crossover produced one offspring. Each of the elements of *P* can be chosen to participate in a tournament of two, implementing the no-replacement tournament selection [36]. The approach chooses the most fitted element of each tournament to produce the crossover without allowing the same chromosome to be the winner of the two tournaments since the tournaments are repeated until two different elements of *P* are selected. This two-point crossover approach was adopted because it was reported to outperform other conventional crossover operations [37]. It is important to note here that in both cases, all the chromosomes have an equal probability of being picked for a tournament, i.e.,

(5) $$\frac{2z-1}{z\left(z-1\right)}\;,\;\mathrm{if}\;\mathrm{and}\;\mathrm{only}\;\mathrm{if},\;z\geq 3$$

However, the most fitted elements will have a higher probability of being selected in each tournament and, consequently, used for crossover or cloning. A mutation operation (that performs the logical not operation) was applied to all elements of the chromosome of each *q* according to the mutation probability, *m_prob_*. Therefore, the estimated number of mutations on a given iteration *g* is given by

(6) $$m_{\mathrm{prob}}{}^{\left(g\right)}\left(z-2\right)N_{\mathrm{bits}}$$

where *N_bits_* is the number of bits used to encode the problem. The implemented methodology for the GA follows the convention of starting with high exploration (using a high *m_prob_*) and then progressively changing into exploitation (decreasing *m_prob_* every five generations). It is worth noting that if both mutation and crossover rates are too high, then the GA will head toward random search, while the opposite leads to a hill-climbing algorithm. Hence, a gradual change from exploration to exploitation is more suitable [38].

The two best *p* of each generation were considered elites, ensuring they were moved to the next generation. Subsequently, the performance of each *q* was assessed and stored. *P* (without the two elites) and *Q* were combined and sorted according to the performance scores (i.e., the attained *PM* by the model defined by the chromosome), from most to least fitted, and the best *z* − 2 members were chosen to compose the new *P*. Afterward, the two elites were introduced in *P*, which was then sorted from most to least fitted (according to the performance scores). Subsequently, a new generation started, and the process was repeated until either *g* was equal to *G* or *Pa* was equal to *Pa_max_*.

#### 2.3.2. Particle Swarm Optimization

PSO is a population-based stochastic optimization algorithm that uses agents (called particles), organized in a swarm (*S*), to search for the optimal solution(s) in a (possibly complex) search space. Each particle *p*, in its turn, is a candidate solution for the optimization problem at hand.

The algorithm was initially proposed in 1995 by R. Eberhart and J. Kennedy [39,40]. These authors suggested a collective search strategy where particles consider the best position found by the other particles (in other words, the social information) and its individual best position (also known as the cognitive information) to explore the search space and converge to the optimal solution(s).

In short, PSO can be described in three main steps: (i) initialize the swarm by randomly positioning the particles in the search space; until a stopping criterion is met: (ii) compute, for each particle, its new velocity (*v*) and position (*x*), and (iii) for each particle, when a better solution is found, update the cognitive and social position information. A pseudocode of PSO is shown in Appendix A (Algorithm A2).

It is important to note that the social position information is shared using information links between particles. These information links allow particles to be fully connected and thus share information with every particle in the swarm or create neighbors of particles where the knowledge is restricted to the particles that belong to the same neighborhood.

To optimize the structure and hyperparameters of the deep learning classifier used in this work, a discrete binary PSO [41] variant was used. The velocity of a particle, at every iteration *i* and dimension *d*, was thus updated as follows

(7) $$v_{d}^{\left(i+1\right)}=\omega^{\left(i\right)}v_{d}^{\left(i\right)}+c_{1}r_{1}^{\left(i\right)}\left(p_{d}^{\left(i\right)}-x_{d}^{\left(i\right)}\right)+c_{2}r_{2}^{\left(i\right)}\left(l_{d}^{\left(i\right)}-x_{d}^{\left(i\right)}\right)$$

where *ω* is the inertia weight parameter [42], *c*_1_ and *c*_2_ are the cognitive and social weights, respectively, and *r*_1_ and *r*_2_ are two uniformly distributed pseudorandom numbers. Finally, *p* is the personal best position found by the particle, and *l* is the best position found by the neighboring particles. After computing the velocity of the particles, the position of each particle is changed according to

(8) $$x_{d}^{\left(i+1\right)}=\left\{\begin{array}{ll} 1, & \mathrm{if}\;rand()<\sigma \left(v_{d}^{\left(i+1\right)}\right)\\ 0, & \mathrm{otherwise} \end{array}\right.$$

where *rand*() denotes a pseudorandom number drawn from a uniform distribution on the interval [0, 1] and *σ* the sigmoid function.

The particles were organized in a ring topology, where each particle only shares information with the two immediately adjacent neighborhoods. The rationale behind the choice of this topology is that in a ring topology, the social information flows slowly, which simultaneously slows down the convergence speed. This behavior is important in multimodal complex optimization problems like the one presented in this paper. Having a low convergence rate improves the algorithm’s exploration capabilities and prevents the premature convergence of the algorithm, therefore, reducing the susceptibility of PSO to getting trapped in a local minimum [43,44]. The inertia weight parameter (*ω*), on the other hand, was updated following a negative non-linear time-varying approach.

### 2.4. Performance Metrics and Validation Methodology

The performance in the experimental results was assessed by the Accuracy (*Acc*), Sensitivity (*Sen*), and Specificity (*Spe*) of the predictions against the ground truth (database labels) by [45]

(9) $$Acc=\frac{TP+TN}{TP+TN+FP+FN}$$

(10) $$Sen=\frac{TP}{TP+FP}$$

(11) $$Spe=\frac{TN}{TN+FN}$$

where *TP* is the number of instances of class “A” classified as class “A”, *TN* is the number of instances of class “not-A” classified as class “not-A”, *FP* is the number of instances of class “not-A” classified as class “A”, and *FN* is the number of instances of class “A” classified as class “not-A”. The diagnostic ability of the algorithm was evaluated by the Area Under the receiver operating characteristic Curve (AUC) [46], considering that the positive class was “A”.

The normalized diversity of the population or particles at each generation or iteration (distance-based measure) was computed as [38,47]

(12) $$Div\left(g\right)=\frac{2}{zL\left(z-1\right)}\overset{z-1}{\underset{\mu =1}{\sum }}\overset{z}{\underset{\theta =\mu +1}{\sum }}Ham\left(p_{\mu },p_{\theta }\right)$$

where *L* is the length of the chromosome or particle, *z* is the number of chromosomes or particles, and *Ham* is the Hamming distance, given by the number of positions where the bits of the two chromosomes differ.

Since the optimization procedure is considerably time-consuming, Two-Fold Cross-Validation (TFCV) was used to find the optimized solution with a cold start of the classifier in each run. TFCV was performed by dividing the subjects into two datasets (ensuring subject-independent datasets by using the data from each subject exclusively in only one of the datasets). The AUC of the two TFCV cycles was averaged to find the mean AUC considered as the PM for the model under examination. The Adam algorithm [48] was used for training since it was found to be the most suited for the CAP analysis based on LSTM [31]. Cost-sensitive learning was employed to deal with the substantial data imbalance (instead of using a balancing operation that can alter the expected distribution of the data) since, for some subjects, more than 80% of the epochs can refer to the “not-A” class.

When the best structure of the classifier was found, the Leave One Out (LOO) method was used to assess the model’s performance, with a cold start (the classifier weights were randomly initialized to not perform retraining) of the classifier in each run. This method was employed as it can provide less biased results when few samples are available [49]. Hence, a total of 16 evaluation cycles were executed. The training set employed for each cycle was composed of data from 15 subjects, and the data from the left-out subject composed the testing set. Each subject was only chosen once to compose the testing set.

### 2.5. Implementation

A resampling procedure was applied to attain a uniform database since the sampling frequency of the records varies between 100 Hz and 512 Hz. All signals were resampled at the lowest sampling frequency by decimation [50]. A constant reduction factor was employed for the sampling rate, *s*, and a standard lowpass filter (Chebyshev type I filter with order eight, normalized cut-off frequency of 0.8/*s*, and passband ripple of 0.05 dB) was used to avoid aliasing and down-sample the signal. Thus, a resampling process chooses each *s*^th^ point from the filtered signal to generate the resampled signal. This signal was then standardized by subtracting the mean and dividing the result by the standard deviation to reduce the effect of systematic variations in the signal [51].

Several studies recommended removing artifacts related to the cardiac field and eye movements during sleep as an approach that can marginally improve the classifier’s performance [52,53]. Nevertheless, the accurate removal of these artifacts requires, at least, the electrooculogram and electrocardiogram signals, leading to a further complex model. Therefore, these artifacts were not removed.

Epoch’s duration (*D*) was selected to be one second, which is in line with the standard duration for CAP analysis, and it corresponded to the database labels. Since the signals were resampled at 100 Hz, the input dimension was 100 for each time step.

For this work, AUC was selected as the PM since it can estimate the diagnostic ability of the algorithm without being significantly affected by class imbalance. For both studied optimization algorithms, the used learning rate was 0.001, and the batch size was 1024. The optimal classification threshold for the test dataset of the LOO examination was identified by finding the optimal cut-off point of the receiver operating characteristic curve estimated on the training dataset.

The optimization algorithm assessed four activation functions to introduce nonlinearities in the network: tanh, sigmoid, Rectified Linear Unit (ReLU), and Scaled Exponential Linear Unit (SELU).

An encoding array, presented in Table A1 (shown in Appendix B), was employed to perform the optimization search. A total of 15 coded chromosomes or particles, each composed of 15 bits, were employed to characterize the population (*P* elements) at each generation or iteration, *g*, using the decoding indicated in Table A1.

For GA, the quality of the solution (fitness assessment) for each element of the population was assessed by the average AUC (employed optimization metric since it reveals the diagnostic ability of the model) estimated by TFCV. The values of *G* and M were chosen to be 20 and 15, respectively. The crossover probability was 90%. The initial mutation probability was 20%, and the value was decreased by 30% every five generations until a minimum of 1% was reached. The GA parameters were selected to be in line with the ones employed by Largo et al. [54], reported as suitable for CAP analysis using a GA.

To allow a fair comparison with GA, a total of 15 particles were employed with PSO (with the same encoding array defined in Table 1), besides keeping the fitness assessment and the stopping criterion as defined previously for GA. Concerning the specific PSO parameters, *c*_1_ was set to 0.6 and *c*_2_ to 0.3 to lead to the convergence of PSO, considering the inertia values [55]. The initial and final values of *ω* were defined as 0.9 and 0.4, respectively [56,57]. To have the same rate of change as the mutation operation in the GA, the value of *ω* was decreased by 9% every five generations until the minimum value of 0.4 was reached.

**Figure 1:**
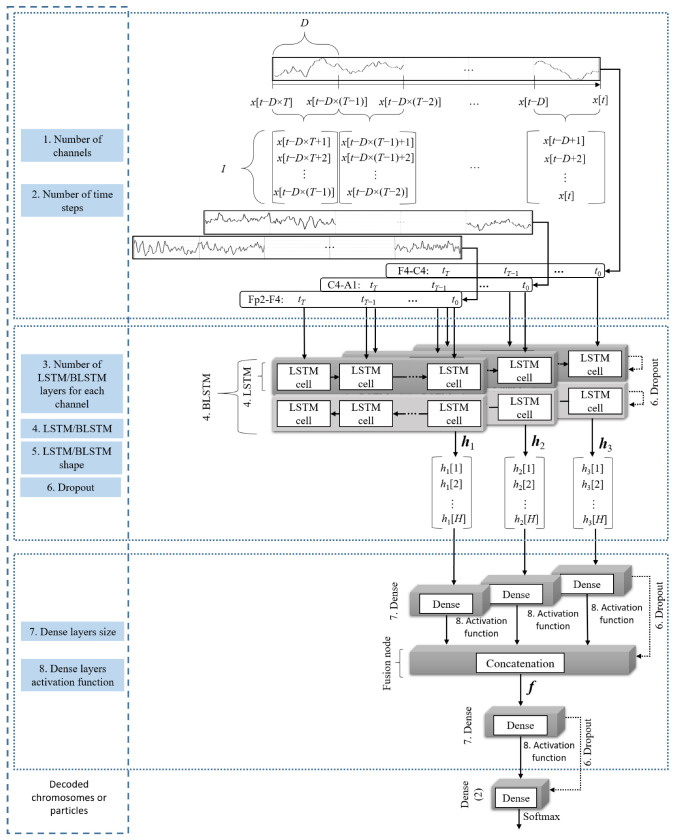
Overview of the implemented model fusing the signal of three EEG channels, using a dense layer to transform the LSTM and concatenation layers outputs.

An overview of the implemented model is presented in Figure 1, where each time step is composed of 100 data points. Since binary classification was employed, an epoch was considered misclassified when the predicted label was bounded by two opposite classifications, denoting an isolated classification. Therefore, in the post-processing, a sequence of 010 was corrected to 000 and 101 to 111.

## 3. Experimental Results

The algorithms were developed in Python 3 using TensorFlow’s libraries to implement the classifier, running in NVIDIA’s GeForce GTX 1080 Ti graphics processing unit. The first step was the search for the best structure of the classifier, performed by the optimization algorithms using TFCV. For the classifiers whose structure was found to be the best by the optimization algorithms, a second performance assessment was carried out by LOO (with a cold start of the classifier in each run).

### 3.1. Optimization of the Classifier

**Figure 2:**
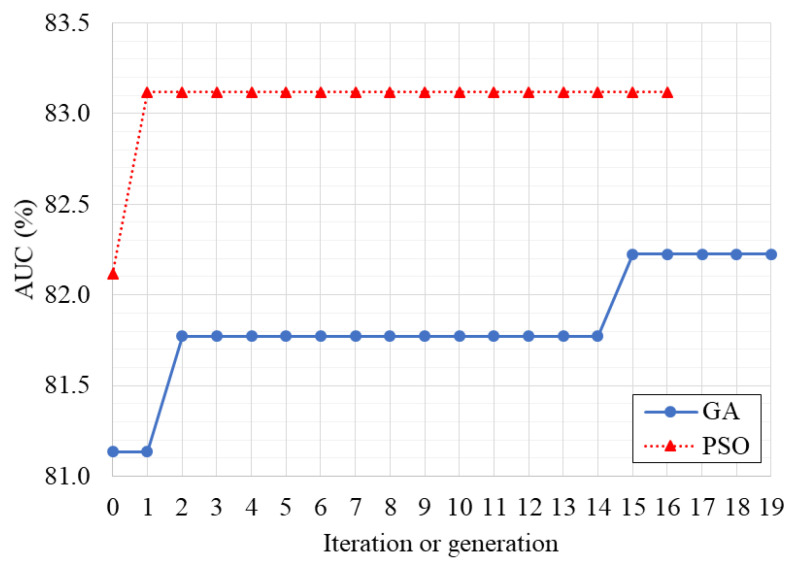
Variation of the AUC of the best solution found by the optimization algorithms.

**Figure 3:**
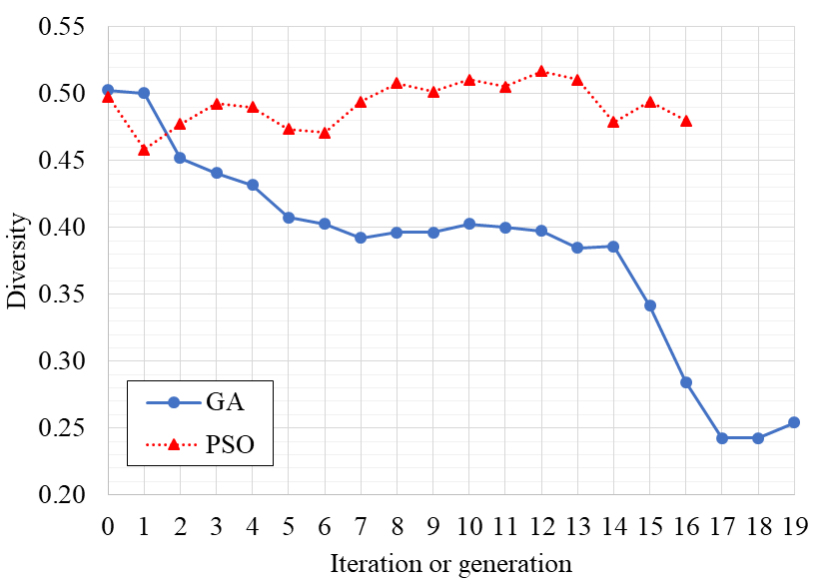
Diversity of the chromosomes or particles over the optimization algorithms’ iterations.

The optimal parameters found by the optimization algorithms are presented in Table 2. Figure 2 and Figure 3 present the AUC variation and the diversity of the chromosomes or particles through the evaluated generations or iterations, respectively. The simulation time was 1,058,067 s (12.25 days) and 859,373 s (9.95 days) for the GA and PSO algorithms, respectively. A total of 300 different networks were simulated by GA, while PSO simulated 255 different networks. It is noteworthy that if a full grid search methodology was employed, the total number of examined networks would be 28,672, which is computationally infeasible.

It was observed in Table 2 that both optimization algorithms identified a similar optimal structure, using the three EEG channels, a single BLSTM layer for each channel with the same shape, and employing the dense layers (one after each BLSTM layer and one after the concatenation layer). On the other hand, the chosen number of time steps was 25 for PSO, being relatively higher when compared to GA (that was 10), with a 10% lower dropout. The selected size and activation function for the dense layer was also different. The total number of trainable parameters was 934,202 and 723,602 for GA and PSO, respectively.

PSO found the best solution at the second iteration, early stopping at iteration 16 (see Figure 2). However, this could mean that PSO converged prematurely, getting trapped into that local optimum. Nevertheless, it was significantly faster than GA, which reached the best solution at generation 15. PSO also maintained a higher diversity in the population (see Figure 3). These results were expected as PSO is prone to converge faster while GA maintains the cycle of offspring creation that progressively decreases the diversity of the population.

### 3.2. Performance Assessment

**Figure 4:**
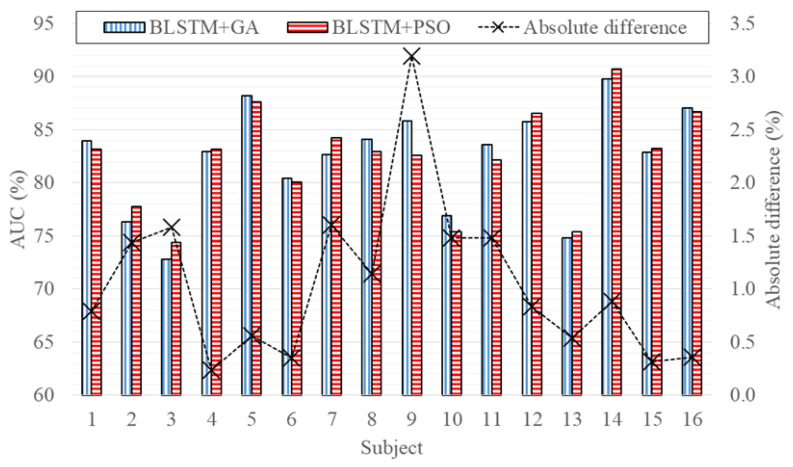
AUC estimation using LOO for the models optimized by GA (BLSTM + GA) and PSO (BLSTM + PSO), depicting the absolute difference between the performance for each examined subject (model evaluating the 16 subjects).

The results obtained by the LOO method using the optimal configurations found by GA and PSO are presented in Table 3, with the 16 subjects; with only the eight subjects FND; with only the eight subjects who have NFLE. Figure 4 depicts the AUC for each subject (subjects 1 to 8 are FND while subjects 9 to 16 have NFLE).

By examining the results from Table 3, when the 16 subjects were used, it is possible to conclude that the configuration found by PSO reached an Acc and Spe, which are approximately 3% and 4% better than the configuration found by GA, respectively. However, the results are less balanced when compared to the configuration found by GA that attained a Sen almost 5% higher. Nevertheless, the AUC of both configurations was approximately the same (82%), indicating that the performance of the two models is equivalent and that both optimization algorithms identified suitable configurations for this analysis. Another relevant aspect, highlighted in Figure 4, is the variation of the performance according to the subjects, demonstrating that the models have an average absolute difference of 1%, and both can work with subjects FND and subjects with NFLE, advocating the feasibility of the proposed model for clinical applications.

When comparing the LOO results (in Table 3) of the models using only the eight subjects FND or only the eight subjects, which have NFLE against the LOO results with the 16 subjects, it is possible to observe that a superior performance for most performance metrics was reached when using LOO with the 16 subjects. These results were expected since the models were optimized to find the best solution when considering a population with both subjects FND and subjects with NFLE. Therefore, the proposed models have the key advantage of being capable of working with both a population FND and a population with sleep disorders (in this case, with NFLE).

### 3.3. Robustness Evaluation

In order to evaluate the robustness of the proposed fusion method, two different tests were performed. The first examined the effect of losing the information from one or two channels, simulating the scenario where some of the electrodes were disconnected (for example, due to movement during sleep). On the other hand, the second test was designed to evaluate the impact of noise on the EEG signals in the model. The models were trained with all channels and without noise. Then, the models were tested by removing channels or introducing noise.

**Figure 5:**
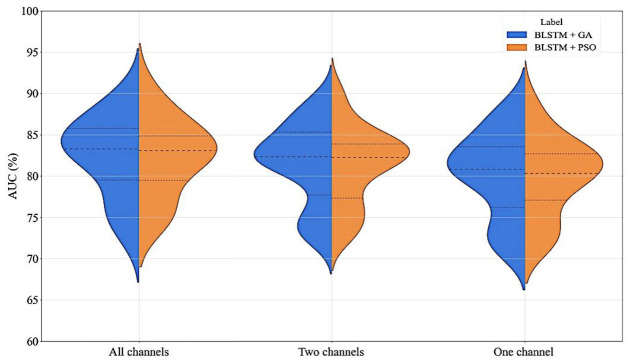
Violin plots of the results attained by LOO when all three channels are available (shown as “All channels”), when one channel failed (shown as “Two channels”), and when two channels failed (shown as “One channel”), for the models optimized by GA (BLSTM + GA, in the left) and PSO (BLSTM + PSO, in the right), depicting the three quartiles (model evaluating the 16 subjects).

The first test results were attained using LOO on the entire population (16 subjects), covering all possible scenarios, and are presented in Figure 5. For the scenario where no channels were lost, indicating three (all) working channels, one channel was lost (shown as two working channels in the figure), and two channels were lost (indicated as one working channel in the figure). The lost channel is replaced by one of the working channels or channel; as for two working channels, it can be replaced by either one of them, whereas for one working channel, all three channels’ inputs are replaced by the remaining channel. By evaluating the results from Figure 5, it is possible to conclude that losing one channel does not considerably change the AUC. Losing two channels (worst case scenario) decreased the AUC median by less than 3% for both models, advocating the robustness of the models.

**Figure 6:**
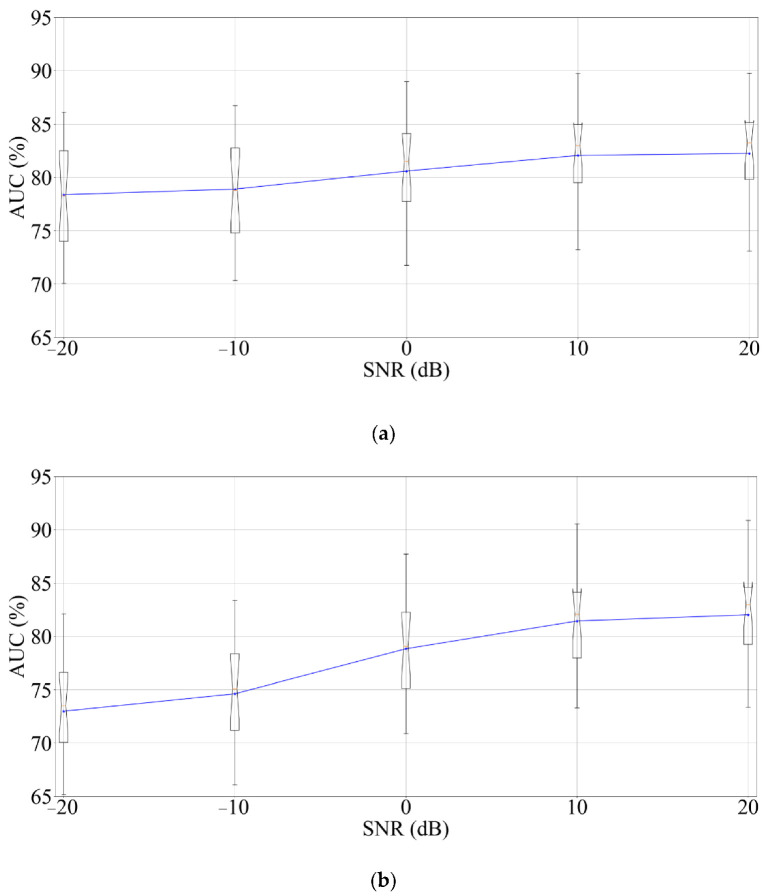
Boxplots indicate the average values for the simulations where AWGN is introduced in the EEG signals, evaluating the 16 subjects using LOO for the models optimized by (**a**) GA, and (**b**) PSO.

To evaluate the effect of having noise in the input signals, all EEG channels were contaminated with Additive White Gaussian Noise (AWGN) with varied Signal to Noise Ratio (SNR) from −20 to 20 dB (range considered suitable for this type of analysis [58]). The results are presented in Figure 6, where it is visible that the model whose structure was selected by GA is less affected by noise than the structure chosen by PSO, conceivably due to the larger number of time steps used by the structure selected by PSO (15 time steps more than the structure selected by GA), which means that more noise will affect the model. Nevertheless, both models maintained a good performance until the SNR was 0 dB, a value considerably lower than the usual SNR of EEG sensors [59]. Therefore, the proposed solutions are also resistant to the introduction of noise in the input channels.

## 4. Discussion

A comparison between the results reported by the previous state-of-the-art works and the results attained in this work is presented in Table 4. By examining the table, it is clear that the previous works that have only studied FNB subjects achieved the best performance, highlighting the difficulties associated with the assessment of subjects with sleep disorders. Although the use of sleep disorder subjects made the classification process more challenging, the produced results can be better generalized for clinical applications.

Another relevant factor is the average number of examined subjects, which was 12 in the state-of-the-art works. In contrast, 18 were examined in this work, emphasizing the viability of the achieved results. It is also important to highlight here the examination of multiple channels considering that, apart from Sharma et al. [60], who evaluated two EEG channels, all state-of-the-art works examined only one EEG channel, which is contrary to the recommendation to score CAP utilizing multiple channels [29], given that an A phase can only be scored if it is visible in all EEG channels. The relevance of using multiple channels is even more emphasized in this work, as both optimization algorithms selected three EEG channels as the best solution.

Contrary to what was done in the developed models, most state-of-the-art works have manually removed the wake or rapid eye movement periods [61,62], which can boost the classifier’s performance. However, it leads to a methodology that is not suitable for implementing a fully automatic scoring algorithm. Additionally, several state-of-the-art works have removed the epochs unrelated to the CAP phase events, lessening the model’s fully automatic applicability [60].

For biomedical applications, it is important to have a balanced performance to provide a reliable clinical diagnosis. Taking into consideration the significant imbalance that characterizes CAP analysis (considerably more events related to “not-A” than “A”), it is not possible to focus the performance assessment only on the Acc since without reporting the Sen and Spe, it is not possible to assess if the performance is balanced or not. Although the AUC is a preferable metric, most of the state-of-the-art works did not report it. Therefore, the mean metric was proposed in this analysis as an alternative to check how balanced the results are. Considering this metric, it is possible to conclude that the best state-of-the-art results, which have included sleep disorder patients in the analysis, are in line with the results attained in this work (76%). However, Mendonça et al. [31,63] examined patients with sleep-disordered breathing while subjects with NFLE were examined in this work. Sharma et al. [60] also evaluated subjects with NFLE but attained a lower Acc, highlighting how difficult it is to examine subjects with this disorder.

It is also important to notice that some state-of-the-art works used a threshold-based approach instead of a machine learning classifier [64,65], which is likely to be difficult to generalize to a broader population. The works based on the manual creation of features to be fed to a classifier also require significant domain knowledge that hampers the research work [20]. Moreover, that methodology usually requires a feature selection procedure to determine the subset of features that are more relevant for the examined problem. On the other hand, the deep learning approach employed in this work automatically creates features. Additionally, the proposed approach can be further improved as more data is available, making the model more suitable for large-scale examinations.

**Table 4:** Comparative analysis between results reported by the state-of-the-art works and the results attained in this work with subjects FND and SDP.

Work	Population (Subjects)	Examined Channel	Acc (%)	Sen (%)	Spe (%)	Mean (%)
[66]	15 FND	C4–A1 or C3–A2	70	51	81	67
[61]	8 FND	C4–A1 or C3–A2	72	52	76	67
[64]	6 FND	C4–A1 or C3–A2	81	76	81	79
[54]	12 FND *	-	81	78	85	81
[67]	4 FND	C4–A1 or C3–A2	82	76	83	80
[68]	15 FND	C4–A1 or C3–A2	83	76	84	81
[65]	10 FND	F4–C4	84	-	-	-
[29]	4 FND	F4–C4	84	74	86	81
[62]	8 FND	C4–A1 or C3–A2	85	73	87	82
[69]	16 FND	C4–A1 or C3–A2	86	67	90	81
[70]	9 FND + 5 SDP	C4–A1 or C3–A2	67	55	69	64
[60]	27 SDP	C4–A1 and F4–C4	73	-	-	-
[63]	9 FND + 5 SDP	C4–A1 or C3–A2	75	78	74	76
[31]	15 FND + 4 SDP	C4–A1 or C3–A2	76	75	77	76
Proposed BLSTM + GA	8 FND +8 SDP	Fp2–F4, F4–C4, and C4–A1	77	73	77	76
Proposed BLSTM + PSO	8 FND +8 SDP	Fp2–F4, F4–C4, and C4–A1	79	68	81	76

* Evaluated one hour of data from each subject.

## 5. Conclusions

A novel methodology to fuse time series signals at the feature level is proposed in this work, and it was evaluated in a challenging real-world scenario of CAP A phase classification. However, this methodology can be used in other contexts when it is intended to fuse information from multiple time series for classification or regression.

The proposed model automatically extracts features by identifying patterns in time from the input time series using a deep learning classifier. However, one of the most challenging aspects of using deep learning models is optimizing the structure and hyperparameters. Two optimization algorithms were examined to address these problems as an efficient alternative to the traditional grid search approach. As a result, it was observed that the optimal structure for the classifier identified by the two optimization algorithms was similar. It selected the input with three EEG signals, denoting the importance of using multiple channels to properly detect the CAP A phases.

It was observed that the obtained performance is in the upper range of the best state-of-the-art works. However, a significantly more challenging methodology of incorporating multiple channels, as well as a more diverse population composed of both FND and NFLE subjects, were used in this work. Contrasting with state-of-the-art, a fully automatic analysis was used instead of manually isolating the NREM sleep epochs. Ultimately, it was observed that the models are resilient to noise and channel failure, making them even more suitable for real-world clinical applications.

It is relevant to notice that the proposed architecture is flexible enough to be altered to include more layers (for example, a combination of convolution layer followed by an LSTM layer instead of only the LSTM layer) or to change the current layers (for example, change the LSTM to a gated recurrent unit).

Three main paths were identified as future work in this research. The first is to validate the proposed methodology further to include more channels in the analysis. The second one is to add different sensors to the fusion model. The last one is implementing a similar methodology to other research and industry applications.

## Figures and Tables

**Table 1:** Demographic characteristics of the studied population.

Measure	Population (Subjects)	Mean	Standard Deviation	Range (Minimum–Maximum)		
Age (years)	8 FND	32.25	5.85	23.00	-	41.00
	8 SDP	33.50	15.04	16.00	-	67.00
	8 FND + 8 SDP	32.88	11.43	16.00	-	67.00
A-phase time (seconds)	8 FND	3235.63	748.10	2235.00	-	4281.00
	8 SDP	4764.75	1069.05	3861.00	-	6901.00
	8 FND + 8 SDP	4000.19	1198.25	2235.00	-	6901.00
REM time (seconds)	8 FND	6997.50	1888.91	4530.00	-	11,430.00
	8 SDP	5703.75	1816.08	2640.00	-	8430.00
	8 FND + 8 SDP	6350.63	1962.53	2640.00	-	11,430.00
NREM time (seconds)	8 FND	20,715.00	2822.07	17,280.00	-	26,040.00
	8 SDP	22,106.25	2459.24	18,210.00	-	26,910.00
	8 FND + 8 SDP	21,410.63	2736.76	17,280.00	-	26,910.00

**Table 2:** Optimal configurations found by the optimization algorithms.

Number	Parameters	Using GA	Using PSO
1	Number of channels to be fused	3 (Fp2–F4, F4–C4, and C4–A1)	3 (Fp2–F4, F4–C4, and C4–A1)
2	Number of time steps to be considered by the LSTM	10	25
3	Number of LSTM layers for each channel	1	1
4	Type of LSTM	BLSTM	BLSTM
5	Shape of the LSTM layers	100	100
6	Percentage of dropout for the recurrent and dense layers	15%	5%
7	Size of the dense layers	300	200
8	Activation function for the dense layers	Sigmoid	ReLu

**Table 3:** Performance attained by the LOO method for the best models identified by the optimization algorithms. Results are presented as “mean ± standard deviation (minimum value–maximum value)”.

Performance Metric	Population (Subjects)	Configuration Found by GA	Configuration Found by PSO
Acc (%)	8 FND + 8 SDP	76.52 ± 4.75 (68.08–85.30)	79.43 ± 4.91 (69.25–87.29)
	8 FND	76.53 ± 4.88 (70.67–87.01)	77.24 ± 6.34 (69.16–86.16)
	8 SDP	77.66 ± 4.55 (71.72–85.91)	79.33 ± 4.74 (71.50–85.35)
Sen (%)	8 FND + 8 SDP	72.93 ± 9.77 (52.64–84.99)	68.14 ± 11.26 (49.36–82.46)
	8 FND	70.04 ± 9.67 (54.86–80.02)	62.79 ± 12.79 (37.60–80.76)
	8 SDP	70.67 ± 12.21 (51.73–85.12)	65.14 ± 14.27 (43.46–85.51)
Spe (%)	8 FND + 8 SDP	77.07 ± 5.96 (66.69–88.12)	81.21 ± 6.71 (68.79–93.35)
	8 FND	77.28 ± 6.05 (69.65–89.22)	79.02 ± 8.40 (67.90–91.95)
	8 SDP	78.69 ± 6.60 (70.83–90.74)	81.90 ± 7.10 (69.83–93.73)
AUC (%)	8 FND + 8 SDP	82.37 ± 4.75 (72.79–89.81)	82.25 ± 4.53 (74.37–90.69)
	8 FND	80.31 ± 4.67 (72.94–87.84)	78.13 ± 3.89 (71.86–83.82)
	8 SDP	82.26 ± 4.75 (74.16–89.52)	81.69 ± 4.96 (74.54–91.10)

## Data Availability

The used data is freely available at the CAP Sleep Database https://physionet.org/content/capslpdb/1.0.0/ (accessed on 3 August 2022).

## Appendix A

**Algorithm A1.** Pseudocode for the GA variant used in this work.

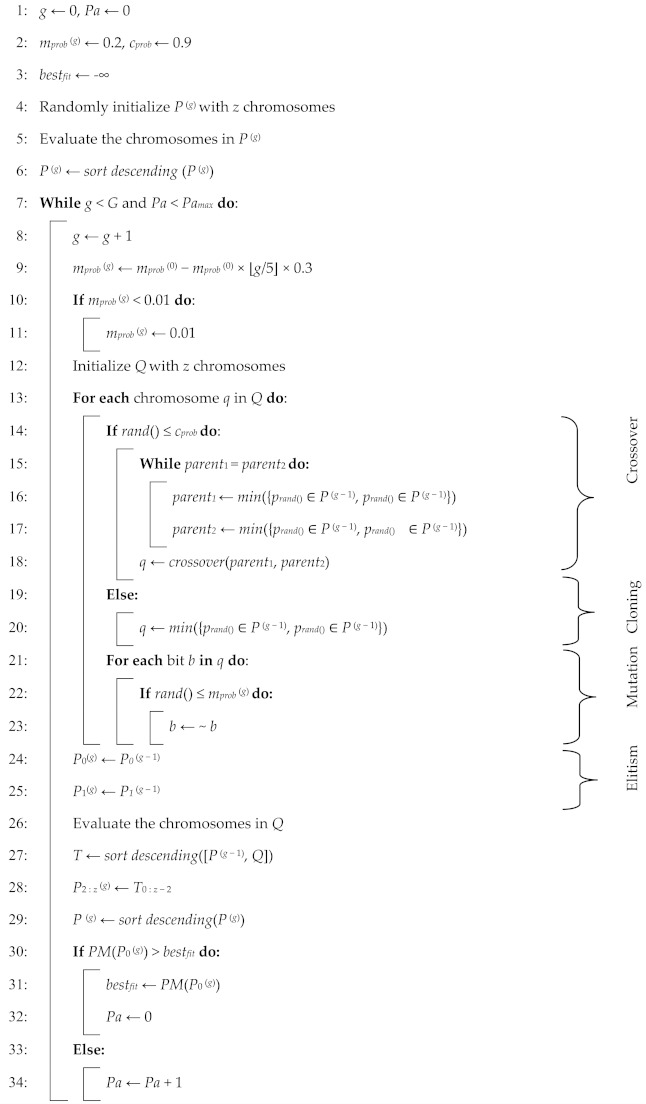

**Algorithm A2.** Pseudocode for the PSO algorithm variant used in this work.

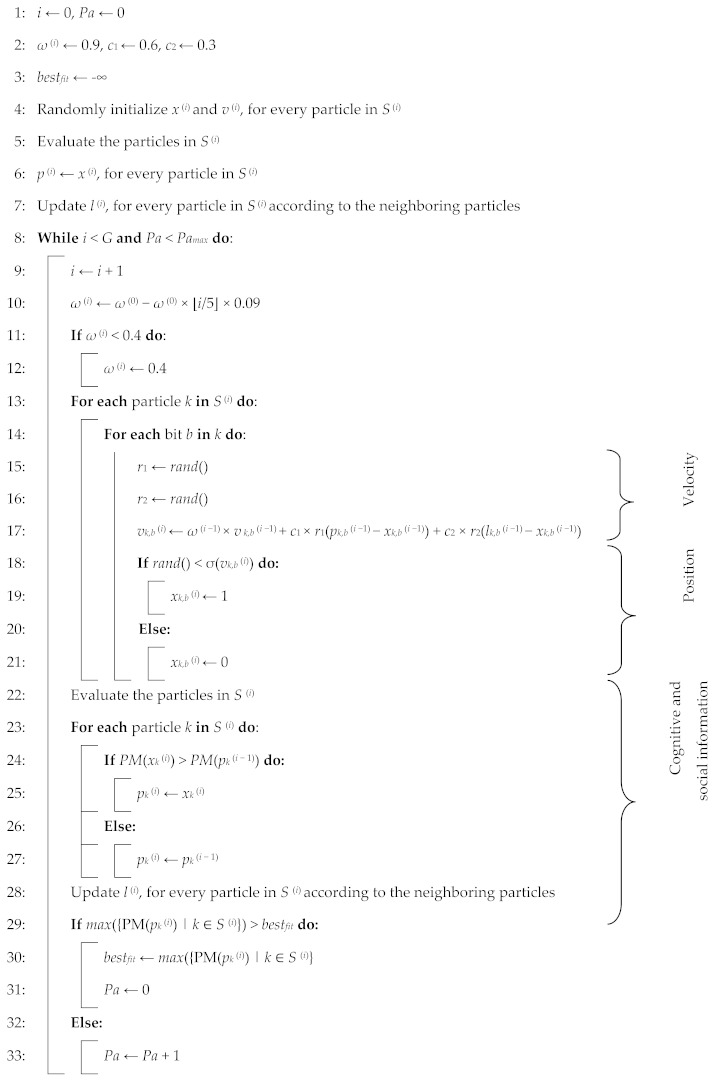

## Appendix B

**Table A1:** Encoding array examined by the optimization algorithms.

Number	Locus	Description	Specification
1	0–2	Number of channels to be fused	000: Fp2–F4 001: C4–A1 010: F4–C4 011: Fp2–F4 and C4–A1 100: Fp2–F4 and F4–C4 101: F4–C4 and C4–A1 110 or 111: Fp2–F4, F4–C4, and C4–A1
2	3–4	Number of time steps to be considered by the LSTM	00: 10 01: 15 10: 20 11: 25
3	5	Number of LSTM layers for each channel	0: One 1: Two staked
4	6	Type of LSTM	0: LSTM 1: BLSTM
5	7–8	Shape of the LSTM layers	00: 100 01: 200 10: 300 11: 400
6	9–10	Percentage of dropout for the recurrent and dense layers	00: 0 01: 5% 10: 10% 11: 15%
7	11–12	Size of the dense layers	00: 0 01: 200 10: 300 11: 400
8	13–14	Activation function for the dense layers	00: tanh 01: Sigmoid 10: ReLU 11: SELU
